# Sotrovimab retains activity against SARS-CoV-2 omicron variant BQ.1.1 in a non-human primate model

**DOI:** 10.1016/j.heliyon.2023.e16664

**Published:** 2023-05-30

**Authors:** Cécile Hérate, Romain Marlin, Franck Touret, Nathalie Dereuddre-Bosquet, Flora Donati, Francis Relouzat, Laura Junges, Mathilde Galhaut, Océane Dehan, Quentin Sconosciuti, Antoine Nougairède, Xavier de Lamballerie, Sylvie van der Werf, Roger Le Grand

**Affiliations:** a Université Paris-Saclay, Inserm, CEA, Center for Immunology of Viral, Auto-immune, Hematological and Bacterial Diseases (IMVA-HB/IDMIT), 18 route du Panorama, 92265, Fontenay-aux-Roses, France; b Unité des Virus Émergents (UVE), Aix Marseille Université, IRD 190, INSERM 1207, 27 Bd Jean Moulin, 13005, Marseille, France; c Institut Pasteur, Université Paris Cité, CNRS UMR 3569, Molecular Genetics of RNA Viruses Unit, 25-28 Rue du Dr Roux, 75015, Paris, France; d Institut Pasteur, Université Paris Cité, National Reference Center for Respiratory Viruses, 25-28 Rue du Dr Roux, 75015, Paris, France

**Keywords:** SARS-CoV2, Sotrovimab, Non human primate, BQ.1.1

## Abstract

The SARS-CoV2 Omicron variants have acquired new Spike mutations leading to escape from the most of the currently available monoclonal antibody treatments reducing the options for patients suffering from severe Covid-19. Recently, both *in vitro* and *in vivo* data have suggested that Sotrovimab could retain partial activity against recent omicron sub-lineage such as BA.5 variants, including BQ.1.1. Here we report full efficacy of Sotrovimab against BQ.1.1 viral replication as measure by RT-qPCR in a non-human primate challengemodel.

## 1 Results

Circulating SARS-CoV-2 Omicron variants have acquired mutations in the receptor-binding domain (RBD) resulting in higher ACE2-binding affinity. These changes are also associated with increasing transmission efficiency and escape to pre-existing neutralizing antibodies. In Europe, the major circulating variant BQ.1.1, derived from BA.5, is escaping most of the available *anti*-SARS-CoV-2 monoclonal antibody treatments. Recent *in vitro* data are controversial with some suggesting that Sotrovimab binds Omicron subvariants, promotes Fc dependent effector functions, and still has the capacity to neutralize them [1]. While most of the *in vitro* studies conducted on cell lines, mainly VeroE6 cells, report a drastic decrease of Sotrovimab inhibition effect on recent omicron subvariant including BQ.1.1 [[2], [3], [4]].

Moreover, treatment of S309 (Sotrovimab parent antibody; 10 or 30 mg/kg) in mice or with Sotrovimab (7 or 14 mg/kg) in hamsters provided protection from a BQ.1.1 challenge [1,5]. However, further data are needed to confirm Sotrovimab *in vivo* activity and pharmacokinetics and to determine whether it should remain, in addition to Nirmatrelvir-Ritonavir in the list of available treatments for patients at risk [6].

In this controversial context and to offer new possibilities to clinicians, we conducted an efficacy study in a well characterized SARS-CoV2 NHP model [[7], [8], [9], [10]]. Here we report Sotrovimab efficacy in a non-human primate (NHP) model of SARS-CoV-2 infection. Three female cynomolgus macaques (*Macaca fascicularis*) aged 14–15 years and weighing between 4.6 and 7.3 kg were treated with 10 mg/kg of Sotrovimab (Xevudy) intravenously 96 h prior to viral challenge. Treated animals and two additional controls were challenged with 1 × 10^5^ pfu of SARS-CoV-2 BQ.1.1 (hCoV-19/France/IDF-IPP50823/2022 - EPI_ISL_15195982) via combined intranasal and intratracheal routes using an experimental protocol previously reported for other variants [10].

**Fig. 1:**
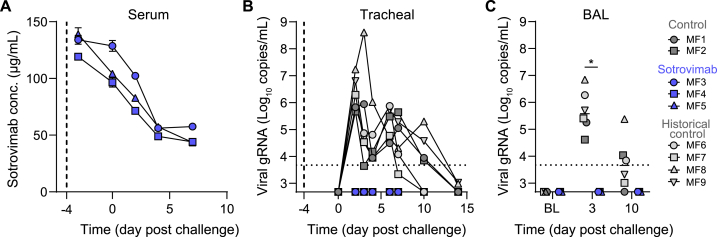
**Pharmacokinetics of Sotrovimab in serum and viral loads in the respiratory tract of BQ.1.1 SARS-CoV-2 exposed cynomolgus macaques treated with Sotrovimab.** Animals MF3, MF4 and MF5 were treated 4 days before challenge (blue) while MF1 and MF2 were not treated (dark grey). MF6, MF7, MF8 and MF9 were added as non-treated historical controls (light grey) **A**. Pharmacokinetics of Sotrovimab in serum. NHPs were challenged 4 days after Sotrovimab injection. **B**. Genomic viral RNA (gRNA) was measured in tracheal fluids collected with swabs during the acute phase of infection. **C.** BAL gRNA was analyzed at baseline, day 3 and day 10 post challenge. Horizontal dotted lines represent the limit of quantification (2.7 log_10_ copies/mL). Vertical dotted lines indicate the day of Sotrovimab treatment. Two-tailed non-parametric Mann-Whitney tests were used for comparisons. *p < 0.05. (For interpretation of the references to colour in this figure legend, the reader is referred to the Web version of this article.)

The treatment was carried out without any adverse effects being recorded. Sotrovimab was measured in the serum of NHP at days 1, 4, 8 and 11 post–treatment, showing a similar exposure profile (Fig. 1A) as observed in humans during the COMET-ICE trial [11]. Lymphopenia was reported at 2 days post-challenge (d.p.c) in non-treated animals and in 2 out of 3 treated animals as expected in this model [10]. In this study, to empower statistical analysis, we included 4 historical controls challenged with the same batch of BQ.1.1 SARS-CoV2 virus stock at a similar dose to the 2 concomitant control NHPs. Efficacy was monitored by genomic viral RNA (gRNA) quantification using RT-qPCR (Fig. 1B). In untreated animals, gRNA was detected in tracheal fluids collected with swabs, with a median peak viral loads at 2–3 d.p.c of 6.11 log_10_ copies/mL. Viral gRNA was also detected at 3 d.p.c in the broncho-alveolar lavages (BAL) with a median value of 5.55 log_10_ copies/mL. At 10 d.p.c, virus was still detectable in BAL in 5 control NHPs out of 6. By contrast, the three treated animals had viral gRNA below the limit of detection both in trachea (Fig. 1B) and BAL (Fig. 1C). Comparisons of the area under the curve (D0-D14) for tracheal viral load kinetics as well as BAL viral load at day 3 reveal a significant difference (p = 0.0238) between controls and Sotrovimab treated animals.

Sotrovimab had previously been withdrawn from the therapeutic panel due to its initially proposed poor *in vitro* efficacy against Omicron variants, in particular BA.2 [12]. Here, we demonstrate that Sotrovimab inhibits viral replication of BQ.1.1 in NHPs both in upper and lower respiratory tracts when given for prophylaxis. Our results support the ability of Sotrovimab to prevent infection with SARS-CoV-2 (10.13039/100011316BQ.1.1 variant) following inoculation therefore it may have potential activity against this variant. Further studies of use of Sotrovimab as a treatment for variant BQ.1.1 are required. Our results thus support the use of Sotrovimab in humans against 10.13039/100011316BQ.1.1, in case of ineligibility to Nirmatrelvir-Ritonavir.

## Author contribution statement

Cécile Herate: Conceived and designed the experiments; Analyzed and interpreted the data; Wrote the paper.

Romain Marlin: Conceived and designed the experiments; Wrote the paper.

Franck Touret; Laura Junges; Mathilde Galhaut; Quentin Sconosciutti: Performed the experiments.

Nathalie Dereuddre-Bosquet: Conceived and designed the experiments; Analyzed and interpreted the data.

Flora Donati; Océane Dehan; Antoine Nougairède: Analyzed and interpreted the data.

Francis Relouzat: Conceived and designed the experiments; Analyzed and interpreted the data.

Xavier de Lamballerie; Sylvie van der Werf; Roger Le Grand, P.h.D, D.V.M: Conceived and designed the experiments.

## Data availability statement

Data will be made available on request.

## Additional information

No additional information is available for this paper.

## Declaration of competing interest

The authors have declared that no conflict of interest exists.

## Appendix

### Materials and methods

#### Antibody treatment

Sotrovimab (Xevudy 500 mg solution to dilute for perfusion) is a commercial antibody also named VIR-7831 and GSK4182136. Recommendations for humans consists in 500 mg by intravenous route, equivalent to 10 mg/kg for a patient of 50 kg. For NHPs, the treatment was administrated 96 h prior challenge by intravenous perfusion in 30 min. Sotrovimab was diluted at 1.434 mg/mL in NaCl 0.9% and injected at a dose of 10 mg/kg, which represents a volume between 34 and 50 mL.

Animals were monitored for heart rate, respiratory rate and oximetry every 10 min from treatment initiation until 30 min after the end of injection.

#### Quantification of Sotrovimab monoclonal antibody

Sotrovimab exposure was measured using the commercial enzyme-linked immunosorbent assay (ELISA) *anti*-SARS-CoV-2 Quantivac (IgG) kit (Euroimmun) which is directed against the S1 domain of the spike protein. Results were expressed in binding antibody units per mL (BAU/mL) following manufacturer instructions and converted to μg/mL using blank plasma from untreated/infected animals spiked with known quantities of Sotrovimab.

#### SARS-CoV-2 challenge

Treated and control animals were challenged with SARS-CoV-2 BQ.1.1 (hCoV-19/France/IDF-IPP50823/2022 - EPI_ISL_15195982), provided by the National Reference Center for respiratory viruses at Institut Pasteur, via the combination of intranasal (1/10) and intratracheal (9/10) routes (day 0), using atropine (0.04 mg/kg) for pre-medication and ketamine (5 mg/kg) with medetomidine (0.05 mg/kg) for anesthesia. Tracheal fluids and blood samples were regularly collected following challenge. Broncho alveolar lavages (BAL) were performed at 3 and 10 days post challenge. NHPs were followed for behavior assessment and clinical score during the 7 days of the infection. Blood cell counts, hemoglobin and hematocrit were determined from EDTA blood using a DXH800 analyzer (Beckman Coulter).

#### Viral quantification

Viral genomic RNA (gRNA) was quantified in swabs and BAL samples by RT-qPCR with a plasmid standard concentration range^.^ The protocols describing the procedure for the detection of SARS-CoV-2 is available on the WHO website (https://www.who.int/docs/default-source/coronaviruse/real-time-rt-pcr-assays-for-the-detection-of-sars-cov-2-institut-pasteur-paris.pdf?sfvrsn=3662fcb6_2).

#### Statistical analysis

Data were collected using classical Excel files (Microsoft Excel 2016). Differences between unmatched groups were compared using the Mann–Whitney *U* test (Graphpad Prism 8.0).
